# Review and prospect of immune checkpoint blockade therapy represented by PD-1/PD-L1 in the treatment of clear cell renal cell carcinoma

**DOI:** 10.32604/or.2023.027942

**Published:** 2023-05-24

**Authors:** WENFEI GE, SHIYAN SONG, XIAOCHEN QI, FENG CHEN, XIANGYU CHE, YONGHAO SUN, JIN WANG, XIAOWEI LI, NANA LIU, QIFEI WANG, GUANGZHEN WU

**Affiliations:** 1 Department of Urology, The First Affiliated Hospital of Dalian Medical University, Dalian, 116011, China; 2 Department of Urology, The First Hospital of Qinhuangdao, Qinhuangdao, 066000, China; 3 Department of General Surgery, The First Affiliated Hospital of Dalian Medical University, Dalian, 116011, China

**Keywords:** RCC, Immunotherapy, PD-1/PD-L1

## Abstract

As a common tumor of the urinary system, the morbidity and mortality related to renal carcinoma, are increasing annually. Clear cell renal cell carcinoma (CCRCC) is the most common subtype of renal cell carcinoma, accounting for approximately 75% of the total number of patients with renal cell carcinoma. Currently, the clinical treatment of ccRCC involves targeted therapy, immunotherapy, and a combination of the two. In immunotherapy, PD-1/PD-L1 blocking of activated T cells to kill cancer cells is the most common treatment. However, as treatment progresses, some patients gradually develop resistance to immunotherapy. Meanwhile, other patients experience great side effects after immunotherapy, resulting in a survival status far lower than the expected survival rate. Based on these clinical problems, many researchers have been working on the improvement of tumor immunotherapy in recent years and have accumulated numerous research results. We hope to find a more suitable direction for future immunotherapy for ccRCC by combining these results and the latest research progress.

## Introduction

Kidney cancer is a common tumor of the urinary system. In 2020, the incidence of renal cell carcinoma was approximately 430,000 cases, accounting for 2.2% of new cancer cases worldwide, and with mortality rate of approximately 180,000 cases, accounting for 1.8% of global cancer deaths [1]. Incidence and mortality rates are increasing every year, posing serious health risks. Renal cell carcinoma (RCC) is the most common form of kidney cancer, accounting for 90% of all cases, and the disease includes over 10 histological and molecular subtypes, with ccRCC being the most common histological type (75%) and the cause of most cancer-related deaths [2,3].

In renal clear cell carcinoma, cytokines are the standard of care for advanced CCRCC before the introduction of targeted vascular endothelial growth factor (VEGF) therapy [4]. However, CRCC is a highly vascular tumor, and in the last 20 years, tyrosine kinase inhibitors (TKI) and anti-angiogenic agents targeting the VEGF pathway have been found to benefit patients with CCRCC, including targeted rapamycin inhibitors (MTOR) as well [5–7]. However, RCC is genetically heterogeneous, and from a long-term perspective, anti-angiogenic drugs are not particularly effective, and patients do not derive long-term anti-cancer benefits due to toxicities and drug resistance. This means that many signaling pathways are involved in regulating tumor growth, not just the mTOR signaling pathway [8].

Immune checkpoint inhibitors (ICI): the PD-1/PD-L1 pathway plays an important role in tumor immunity [9], and the combination of PD-1 and PD-L1 acts as a suppressor of the host’s anti-tumor immunity, leading to tumor immune escape. Based on this mechanism, various types of anti-PD-1/PD-L1 antibodies (α-PD-1/PD-L1) are used to treat cancers, including renal cell carcinoma. However, the low response rate to treatment and toxic side effects of these drugs remain to be addressed. As anti-PD-1/PD-L1 antibody drugs indirectly enhance T-cell reactivity and effector function, leading to the development of autoimmune diseases, PD-1/PD-L1 blockade therapies face many challenges in producing more beneficial clinical outcomes in patients. The combination of anti-PD-1 or anti-PD-L1 therapy with other therapeutic modalities may be the main option to achieve this, where the combination of VEGFR or mTOR inhibitors with ICI and geotechnically improves the prognosis of patients with renal cell carcinoma.

The advent of ICI has benefited countless patients; however, due to its ineffectiveness in unscreened patients and the presence of side effects, exploring how to use a combination of tumor cell death and the host’s own immune system to remove tumor cells may be necessary and a viable and effective therapeutic strategy. ICD can cause cell death in the immune response and initiate a T cell-mediated adaptive immune response, resulting in long-term tumor suppression, and the study and exploration of the ICD mechanism may provide direction for the next step in treatment.

## Development of Targeted Cancer Therapies

### Oncolytic virotherapy

The earliest examples of cancer immunotherapy date back to 1891, when the first attempts to use the immune system to treat cancer were made, after it was noted that a mixture of live and inactivated *Streptococcus pyogenes* and *Serratia marcescens* could lead to tumor regression in patients with sarcoma [10,11]. Decades later, the use of genetically modified viruses to infect tumor cells and stimulate a pro-inflammatory environment, thereby enhancing systemic anti-tumor immunity, was known as oncolytic virotherapy [12,13]. With advances in genetic engineering and viral transformation techniques, oncolytic virotherapy has made significant progress in recent years. Talimogene laherparepvec (T-Vec), also known as Imlygic, a genetically modified herpes simplex virus, has shown promising clinical benefits in patients with advanced melanoma and has been approved for the treatment of unresectable metastatic melanoma [14,15]. Moreover, T-Vec plus pembrolizumab treatment has a manageable safety profile associated with antitumor activity in advanced sarcomas of a range of sarcoma histological subtypes [16].

### Cancer vaccines

There are three main categories of cancer vaccines: cellular vaccines (tumor or immune cells), protein/peptide vaccines, and nucleic acid vaccines (DNA, RNA, or viral vectors) [17]. The key research comes from the identification of both melanoma-derived antigens encoded by the MAGE (melanoma associated antigen) gene family, MZ2-E and MZ2-D, which are recognized by cytotoxic T cells to trigger an anti-tumor immune response [18,19]. To date, tumor vaccines have been tested for a variety of tumor treatments, including melanoma [20–22], lung cancer [23,24], renal cell carcinoma [25], and prostate cancer [26]. In addition to tumor antigens, DC-based vaccines have shown significant clinical results. Nucleic acid vaccines may be promising for several reasons. Unlike peptide vaccines, nucleic acid vaccines can encode full-length tumor antigens, allowing APCs to simultaneously present or cross-present multiple epitopes with class I and class II patient-specific human leukocyte antigens (HLA). Therefore, they are less restricted by human HLA and more likely to stimulate a broader range of T-cell responses [27]. To date, there are more than 20 HLA-based antibodies and more than 20 mRNA-based immunotherapies have entered clinical trials, some of which have demonstrated the feasibility of this therapy [28]. Sahin conducted a phase I trial (Lipo-Merit trial, ClinicalTrials.gov identifier NCT02410733) in patients with advanced melanoma testing melanoma FixVAC (BNT111), an intravenous liposomal RNA (RNA-LPX) vaccine. The results showed that RNA-LPX vaccination is an effective immunotherapy for patients with CPI-type melanoma [29]. However, intrinsic tumor cell resistance and local or systemic immunosuppression (extrinsic) mechanisms greatly compromise the efficacy of cancer vaccines, allowing the body to develop resistance, resulting in less effective cancer treatment.

### Cytokine therapy

As messengers that coordinate cellular interactions and communication in the immune system, cytokines are major regulators of the innate and adaptive immune system, allowing the immune cells to communicate over short distances in a paracrine and autocrine manner. The main cytokines involved in cellular communication in homeostasis and disease are interleukins (IL), including IL-2, IL-7, IL-12, and IL-15, interferons (IFN), members of the tumor necrosis factor (TNF) superfamily, chemokines (chemotactic cytokines), and growth factors. Considering the ability of the immune system to recognize and destroy cancer cells, cytokines have been used to treat cancer [30–35] for more than 40 years, initially, owing to the identification of IL-2 [36]. During the early 1990s, clinical trials of high-dose IL-2 in patients with metastatic renal cell carcinoma and malignant melanoma showed that some patients with metastatic renal cell carcinoma and metastatic melanoma benefited from high-dose IL-2 treatment [37,38]. In addition to IL-2, other cytokines have also been used in cancer treatment, such as INF-α in the treatment of chronic granulocytic leukemia, malignant melanoma, and renal cell carcinoma [4,39,40]. Although many clinical trials have demonstrated the effectiveness of cytokines in cancer treatment to varying degrees, factors such as toxic side effects and poor patient tolerance suggest that single cytokine therapy is not the best clinical treatment option.

## Advances in the Treatment of CCRCC

### Targeted therapy

**Figure 1:**
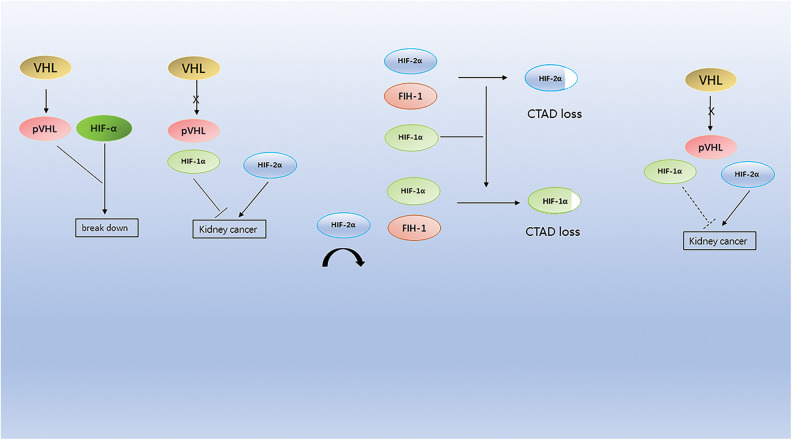
Mutations in the VHL gene lead to the accumulation of HIF-α protein, which, because HIF-1α is more sensitive to FIH-1 compared to HIF-2α, leads to the accumulation of HIF-2α in cells and eventually to kidney cancer.

In renal clear cell carcinoma, cytokines, including high-dose IL-2 and IFN-α, were the standard of care for advanced CCRCC before the introduction of targeted vascular endothelial growth factor (VEGF) therapy [4]. However, CRCC is a highly vascular tumor. Inactivation of the von Hippel Lindau (VHL) tumor suppressor gene is a characteristic lesion in renal clear cell carcinoma (The mutations associated with kidney cancer are shown in Table 1), leading to overexpression of the hypoxia-inducible factor (HIF)-2α oncoprotein and its downstream targets, including (VEGF) [41] (Fig. 1).

**Table 1:** Genes associated with kidney cancer

Gene	The influence of metabolic pathways	Association with kidney cancer	References
VHL	Inhibits HIF-1a, HIF-2a activity and regulates anaerobic glycolysis	Mutations in the VHL gene can lead to the accumulation of hif-2α and subsequently to kidney cancer.	[41]
PBRM1	Maintaining the stability of chromosomes during mitosis and participating in the regulation of the cell cycle	High mutation rate in kidney cancer	[41,42]
BHD	Binds to AMPK, regulates the mTOR pathway and is involved in cell growth, proliferation, differentiation and cell cycle regulation	Expression of multiple mTOR-related proteins is upregulated in kidney cancer	[41,43]
TGF-β1	Inhibition of proliferation via the classical Smad signaling pathway	Mutation of TGFBR2/TGFBR1 leads to inhibition of the smad pathway. TGF-β1 expression was significantly elevated in renal cell carcinoma tissue	[44]
SETD2	Participates in histone methylation modifications and acts with RNA polymerase II to mediate transcriptional elongation and mismatch repair	Post-mutation affects the prognosis of kidney cancer	[45]

As a result, tyrosine kinase inhibitors (TKI) and anti-angiogenic drugs targeting the VEGF pathway have been shown to benefit patients with CCRCC in clinical trials, including targeted rapamycin inhibitors (MTOR) [5–7]. Over the past 20 years, various antiangiogenic drugs targeting the VEGF receptor, including axitinib, pazopanib, sunitinib, and sorafenib, have been shown to be effective in phase 3 clinical trials [6,46,47]. However, for a long-term perspective, antiangiogenic drugs are not particularly effective because of their toxic side effects and drug resistance.

mTOR is a serine/threonine kinase and a complex of two distinct proteins, mTORC1 and mTORC2. Inhibition of the mTOR pathway affects cellular functions including cell growth, proliferation, metabolism, and angiogenesis. The mTOR pathway gene expression levels can influence targeted drug therapy in most CCRCCs [48] and therefore provide therapeutic ideas for different types of cancer, including advanced renal cell carcinoma [49,50]. For example, sirolimus inhibits the mTOR pathway gene expression in most CCRCC. For example, sirolimus inhibits the mTOR pathway, and a phase 3 clinical trial demonstrated that patients treated with tesilimus had a longer overall survival compared to the INF-α group [5]. Everolimus also inhibits the mTOR pathway. Motzer et al. found in a phase 3 randomized, double-blind, placebo-controlled trial that everolimus treatment could prolong progression-free survival in patients with metastatic renal cell carcinoma compared to the placebo group [51]. Although inhibition of the mTOR signaling pathway is beneficial in the treatment of RCC, patients do not derive long-term anti-cancer benefits from it [8]. This means that many signaling pathways are involved in regulating tumor growth, not just mTOR signaling.

### ICI

The PD-1/PD-L1 pathway plays an important role in tumor immunity [9]. As the tumor grows, the combination of PD-1 and PD-L1 inhibits the host’s anti-tumor immunity, which leads to tumor immune escape. Renal cell carcinomas are highly immunogenic, with PD-L1 overexpressed in approximately 30% of renal cell carcinomas [52]. Based on this mechanism, various types of anti-PD-1/PD-L1 antibodies (α-PD-1/PD-L1) are used in the treatment of cancers, including renal cell carcinoma, such as anti-PD-1 antibodies (nivolumab, pembrolizumab, emiplimab) and anti-PD-L1 antibodies (atezolizumab, avelumab, and duravulumab) (Table 2).

**Table 2:** ICIs

Medicine	Target	Effectiveness in cancer	Effectiveness in treating kidney cancer (compared to sunitinib)	References
Nivolumab	PD-1	Metastatic melanoma, metastatic non-small cell lung cancer, advanced renal cell carcinoma, metastatic squamous cell carcinoma, metastatic colorectal cancer, hepatocellular carcinoma	Higher OS, PFS, ORR, CR in combination with cabozantinib	[53]
Pembrolizumab	PD-1	The most widely indicated ICI in the world, with 17 indications in 11 tumor types, including: melanoma, non-small cell lung cancer, head and neck squamous cancer, classical Hodgkin’s lymphoma, uroepithelial cancer, gastric cancer, etc.	Significant prolongation of PFS, OS in combination with axitinib. OR, CR, PD extension	[54]
Ipilimumab	CTLA-4	Melanoma, renal cell carcinoma, metastatic colorectal cancer	Higher OS, PFS, ORR, CR in combination with nivolumab	[55]
Avelumab	PD-L1	Metastatic Merkel cell carcinoma, locally advanced or metastatic uroepithelial carcinoma, advanced renal cell carcinoma	Higher PFS, ORR, CR in combination with axitinib	[56]
Atezolizumab	PD-1	Uroepithelial carcinoma, NSCLC, triple negative breast cancer, small cell lung cancer	Prolonged PFS with combination bevacizumab treatment	[57]

In several clinical trials, they have been shown to play an active role in the treatment of cancer, including renal cell carcinoma.

### α-PD-1/PD-L1

Nivolumab is an immunoglobulin-G4 and PD-1 immunoblocker antibody that blocks contact between PD-1 and PD-L1 only. This therapy holds great promise and has been approved for metastatic melanoma and non-small-cell lung cancer [58,59]. In a phase 3 clinical trial, Motzer et al. randomized 821 patients with advanced clear cell renal cell carcinoma previously treated with anti-angiogenic drugs (in a 1:1 ratio) to receive 3 mg/kg of nabritumomab intravenously every 2 weeks and 10 mg of everolimus tablets orally, once daily. The results showed that the median overall survival was greater in the nabugliumab group than in the everolimus group, the risk of death ratio for nabugliumab *vs*. everolimus was 0.73, the objective remission rate was higher in the nabugliumab group than in the everolimus group, and the median progression-free survival was greater in the nabugliumab group than in the everolimus group. In conclusion, nabugliumab has a longer overall survival, provides a more durable treatment response, and has a more controlled safety profile than everolimus in previously treated patients with advanced renal cell carcinoma [60].

Pembrolizumab is a high affinity humanized IgG4 κ-anti-PD-1 antibody. In a clinical trial, McDermott et al. received pembrolizumab 200 mg intravenously every 3 weeks for ≤24 months in ccRCC patients who had not previously received systemic therapy. The ORR was 26.7% in all enrolled patients. Median duration of response was 29.0 months; 59.7% of responses lasted ≥12 months, median progression-free survival was 4.2 months; 24-month survival rate was 18.6%, and median overall survival was 28.9 months. This clinical trial demonstrated that single-agent pembrolizumab resulted in lesion reduction in the majority of patients with advanced CCRCC, suggesting that it showed effective antitumor effects as first-line treatment [61,62]. In the phase 3 KEYNOTE-564 trial, investigators recruited patients (aged ≥18 years) with CCRCC who were at an increased risk of recurrence. They were administered 200 mg pembrolizumab or placebo (both intravenously) every 3 weeks for 17 cycles. As per the results, disease-free survival was found to be greater with pembrolizumab than with the placebo. This latest result supports the use of adjuvant pembrolizumab monotherapy as the standard of care for patients with renal cell carcinoma following nephrectomy [5].

Atezolizumab blocks PD-L1 expression on the tumor surface and exhibits good antitumor activity. In a phase 1 study, atezolizumab was administered intravenously every three weeks to 70 patients with metastatic RCC. Sixty-two patients had a superior value for objective remission rate (ORR; 15%; 95% CI, 7%–26%), and atezolizumab in renal cell carcinoma patients demonstrated manageability and safety [63], these results will guide relevant clinical studies. However, in a recent phase 3 trial, individual participants were randomly assigned (1:1) to receive intravenous atezolizumab (1200 mg) or placebo once every 3 weeks for 16 cycles or 1 year. The median disease-free survival for atezolizumab was greater than that for placebo [64]. The results of this trial suggest that atezolizumab did not improve clinical outcomes compared with placebo for adjuvant therapy after resection in patients with renal cell carcinoma at an increased risk of recurrence, suggesting that atezolizumab alone is not ideal for the treatment of patients with CCRCC, which may open the way for its use in combination with other agents.

Clinical trial data (Table 3) show that alpha-PD-1/PD-L1 can play a positive role in the treatment of patients with renal cell carcinoma, and some clinical trials have shown that its anti-cancer effects are superior to those of anti-VGEF drugs. Although several drugs have been approved for the treatment of various cancers, the low response rate and toxic side effects of these drugs remain unaddressed. The indirect enhancement of T-cell reactivity and effector function by anti-PD-1/PD-L1 antibody drugs has led to the development of several autoimmune diseases. These side effects include fatigue, skin toxicity (rash and vitiligo), diarrhea, colitis, hepatotoxicity, pneumonia, etc. [76]. Most importantly, the majority of patients do not benefit from the PD-1/PD-L1 blockade during the course of treatment [77]. Theoretically, the exclusion of relevant adverse factors (aberrant angiogenesis [78], cytokines involved in cancer immune escape [79] tumor-associated adipocytes [80] and tumor-associated fibroblasts [81]) could improve the therapeutic efficacy of α-PD-1/PD-L1 and thus increase response rates. Conversely, activation of corresponding favorable factors may promote tumor clearance. For example, one study found that oxidative stress (OS) scores were negatively correlated with CTLA-4 and PD-1 expression, and it can be hypothesized that activation of the OS pathway may inhibit tumor immune escape [82].

**Table 3:** Clinical trial data related to ICI and ICI combined with other drugs in the treatment of RCC

Susbstances	n	Patients	Results			Adverse events	References
Nivolumab *vs*. everolimus phase 3	410 *vs*. 411	Treated,advanced renal cell carcinoma, Karnofsky performance status of ≥70%	PFS(median): 4.6 months *vs*. 4.4 months (*p* = 0.11)	OS (median): 25.0 months *vs*. 19.6 months	ORR(median): 25% *vs*. 5% (*p* < 0.001)	Grade 3 or 4: 19% *vs*. 37%	[60]
KEYNOTE-564 pembrolizumab *vs*. placebo phase 3	496 *vs*. 498	post-nephrectomy,clear cell renal cell carcinoma, no systemic treatment	The disease-free survival rate of pembrolizumab was better than that of placebo (HR 0.63 [95% CI 0.50–0.80]); Estimated proportion of medium survival without recurrence: 75.2% *vs*. 65.5%; Overall survival was better with pembrolizumab compared with placebo (HR 0.52 [nominal 95% CI 0.31–0.86]). Estimated proportion of surviving participants: 95.7% *vs*. 91.4%			Treatment induced serious adverse events: 59 (12%) *vs*. 1 (<1%)	[61,62]
Atezolizumab *vs*. placebo phase 3	390 *vs*. 388	Nephrectomy with or without metastasis,CCRCC or RCC with sarcomatoid component	Median investigator-assessed disease-free survival: 57.2 months (95% CI 44.6 to not evaluable) *vs*. 49.5 months (47.4 to not evaluable; hazard ratio 0.93, 95% CI 0.75–1.15, *p* = 0.50)			Grade 3 or 4: hypertension (7 [2%] *vs*. 15 [4%]), hyperglycaemia (10 [3%] *vs*. 6 [2%]), and diarrhoea (2 [1%] *vs*. 7 [2%])	[63,64]
Nivolumab with cabozantinib *vs*. sunitinib phase 3	323 *vs*. 328	Untreated, advanced renal cell carcinoma with clear cell composition,Karnofsky performance status of ≥70%	PFS(median): 16.6 months *vs*. 8.3 months (*p* < 0.001)	OS (median): 18.1 months The probability of overall survival at 12 months was 85.7% *vs*. 75.6% (*p* = 0.001)	ORR(median): 55.7% *vs*. 27.1% (*p* < 0.001)	Grade 3 or 4: 75.3% *vs*. 70.6%	[65,66]
KEYNOTE-426 pembrolizumab + axitinib *vs*. sunitinib phase 3	432 *vs*. 429	Treatment-naive,advanced renal cell carcinoma with clear cell composition	PFS(median): 15.4 months *vs*. 11.1 months (*p* < 0.0001)	OS (median): not reached *vs*. 35·7 months (*p* = 0.0003)	ORR(median): 60.0% *vs*. 40.0% (*p* < 0.0001)	Grade 3 or 4: Hypertension (95 [22%] *vs.* 84 [20%]), elevated alanine aminotransferase (54 [13%] *vs*. 11 [3%]) and diarrhea (46 [11%] *vs*. 23 [5%])	[67]
Lenvatinib with pembrolizumab *vs*. lenvatinib + everolimus *vs*. sunitinib phase 3	355 *vs*. 357 *vs*. 357	Untreated,advanced renal cell carcinoma, Karnofsky performance status of ≥70%	PFS(median): 23.9 months *vs*. 14.7 months *vs*. 9.2 months (*p* < 0.001)	OS (median): not reached with any treatment; alive at 24 months: 79.2% *vs*. 66.1% *vs*. 70.4%	ORR(median): 71.0% *vs*. 53.5% *vs*. 36.1% complete response(CR): 16.1% *vs*. 9.8% *vs*. 4.2%	All grades: 99.7% (lovatinib pebruzumab group and lovatinib everolimus group) *vs*. 98.5% (sunitinib group) Grade 3 or 4: 82.4% *vs*. 83.1% *vs*. 71.8%	[68,69]
JAVELIN kidney 101 avelumab + axitinib *vs*. sunitinib phase 3	442 *vs*. 444	Untreated, advanced renal cell carcinoma with clear cell composition, 830 patients, including approximately 580 patients with PD-L1–positive tumors (70%), Eastern Cooperative Oncology Group (ECOG) performance-status score of 0 or 1	PFS(median): 13.3 months *vs*. 8.0 months 13.8 months *vs*. 7.0 months (PD-L1–positive) (*p* < 0.0001)	OS (median): Impossible to estimate (NE) *vs*. NE (*p* = 0.0329); NE *vs*. 28.6 months (PD-L1–positive) (*p* = 0.1301)	ORR(median): 52.5% *vs*. 27.3%; 55.9% *vs*. 27.2% (PD-L1–positive)	All grades: 99.5% *vs*. 99.3% Grade 3 or 4: 71.2% *vs*. 71.5%	[70–73]
CheckMate 214 nivolumab + ipilimumab *vs*. sunitinib phase 3	550 *vs*. 446	Untreated, advanced or metastatic renal cell carcinoma with clear cell composition, performance status of ≥70%	PFS (median): 12.3 months *vs*. 12.3 months)	OS (median): 55.7 months *vs*. 38.4 months	ORR(median): 39.3% *vs*. 32.4%	Grade ≥3 immune-mediated adverse event experience, body mass index, and age	[74,75]

## Combination of α-PD-1/PD-L1 with Other Therapeutic Modalities

PD-1/PD-L1 blockade therapies face many challenges in achieving more beneficial clinical outcomes in patients. Anti-PD-1 or anti-PD-L1 therapy in combination with other therapeutic modalities may be the main option to achieve this, including conventional chemotherapy, radiation therapy, targeted therapy, dual immune checkpoint blockade, interferon gene stimulator (STING) agonists, fecal microbial transplantation (FMT), and epigenetic modulators, which contain dozens of combinations of therapeutic modalities, but relevant clinical trials have not The positive results obtained in preclinical trials have not been confirmed in clinical trials [83]. Currently approved combination therapies for cancer treatment include alpha-PD-1/PD-L1 in combination with chemotherapy, angiogenesis inhibitors, and alpha-CTLA-4.

### α-PD-1/PD-L1 in combination with anti-angiogenic drugs

Among them, based on several clinical trials [65,67,70], alpha-PD-1/PD-L1 in combination with angiogenesis inhibitors (including pembrolizumab in combination with axitinib, nivolumab in combination with cabozantinib and avelumab in combination with axitinib) were approved as first-line treatment for renal cell carcinoma.

In a phase 3 trial of nivolumab plus cabozantinib, investigators recruited untreated patients with advanced CCRCC (aged ≥18 years) randomly assigned to receive intravenous 240 mg nivolumab every 2 weeks + 40 mg cabozantinib (once daily) or 50 mg sunitinib (once daily) (4 weeks in 6-week cycles). Results: The median overall survival of 651 patients was 18.1 months; the median progression-free survival was 16.6 months for nivolumab plus cabozantinib compared with 8.3 months for sunitinib. 12-month overall survival was 85.7% in the nivolumab plus cabozantinib group compared with 75.7% in the sunitinib group. 75.6% in the sunitinib group [65]. The overall survival rate at 12 months was 85.7% in the nivolumab plus abozantinib group compared to 75.6% in the sunitinib group. The trial results showed significant benefits in terms of progression-free survival, overall survival, and the likelihood of remission for nivolumab plus cabozantinib compared to sunitinib. In the latest phase 3 trial, investigators randomly assigned (1:1) previously untreated patients (aged ≥ 18 years) with advanced or metastatic renal clear cell carcinoma to receive either 240 mg of intravenous nabulizumab (every 2 weeks), + 40 mg of cabozantinib orally (once daily), or 50 mg of sunitinib orally (once daily) (4 weeks in 6-week cycles). RESULTS: At extended follow-up, median overall survival was 37.7 months in the nabritumomab + cabozantinib group, greater than the 34.3 months in the sunitinib group, with updated median progression-free survival of 16.6 months and 8.3 months in the two groups, respectively [66]. The trial further supports nivolumab plus cabozantinib as a first-line treatment for advanced renal cell carcinoma based on the improved efficacy of nabulizumab + cabozantinib compared to sunitinib, as shown in the extended follow-up and final overall survival analysis.

The first interim analysis of the pembrolizumab plus axitinib KEYNOTE-426 study showed that pembrolizumab plus axitinib was more effective in the treatment of naïve, advanced renal cell carcinoma than sunitinib monotherapy. In the phase 3 KEYNOTE-426 study, investigators recruited untreated patients (aged ≥18 years) with advanced renal cell carcinoma. Patients were randomly assigned (1:1) to receive either 200 mg pembrolizumab intravenously (once every 3 weeks) combined with 5 mg axitinib orally (twice daily) for 35 cycles, or 50 mg sunitinib orally (every 6 weeks) for 4 weeks. RESULTS: The median survival and progression-free survival were better in the pembrolizumab plus axitinib group than in the sunitinib group [67]. These results suggest that pembrolizumab plus axitinib has superior clinical efficacy compared to sunitinib, and the results support pembrolizumab plus axitinib as a first-line treatment for advanced renal cell carcinoma.

Results from the Pembrolizumab plus Lenvatinib phase 3 CLEAR study showed that Pembrolizumab plus Lenvatinib improved progression-free survival and overall survival compared to sunitinib in patients with advanced renal cell carcinoma [68]. In the latest clinical trial, patients were randomly assigned (1:1:1) to 20 mg of levatinib orally (once daily) plus 200 mg of intravenous pembrolizumab (once every 21 days), 18 mg of levatinib orally (once daily) plus 5 mg of everolimus orally (once daily) (21-day cycle), or 50 mg of sunitinib orally (once daily with a 2-week break after 4 weeks). The results showed that patients administered pembrolizumab plus lenvatinib had better outcomes than those treated with sunitinib [69]. These results support the efficacy and safety of pembrolizumab plus lenvatinib as first-line treatment for patients with advanced renal cell carcinoma.

Avelumab plus axitinib showed progression-free survival, overall survival, and objective remission benefits compared to sunitinib in the phase 3 JAVELIN Renal 101 trial in patients with advanced renal cell carcinoma (aRCC) treated with avelumab + axitinib in first line [70–72]. In the latest clinical trial, progression-free survival, objective remission rates, overall survival, and safety were assessed in patients aged <65 years, ≥65 to <75 years, and ≥75 years according to a blinded independent center evaluation (RECIST 1.1), with extended follow-up demonstrating the good efficacy of avelumab plus axitinib in all age groups [73]. The results of the extended follow-up demonstrated that avelumab plus axitinib showed good efficacy in all the age groups. This trial further supports the feasibility of using avelumab plus axitinib as first-line treatment for advanced renal cell carcinoma.

### α-PD-1/PD-L1 in combination with α-CTLA-4

Cytotoxic T-lymphocyte-associated antigen 4 (CTLA-4), a surface receptor on T lymphocytes, can also be used as an immune checkpoint inhibitor to treat cancer, similar to PD-L1. This suggests that renal cell carcinoma can respond to CTLA-4 blockade [84]. The phase 3 trial compared nivolumab plus ipilimumab with sunitinib in previously untreated patients with advanced renal cell carcinoma, in which operators randomized untreated patients with advanced renal cell carcinoma to receive nivolumab (3 mg/kg) plus ipilimumab (1 mg/kg) every 3 weeks for 4 cycles, followed by nivolumab or sunitinib. nivolumab or sunitinib (50 mg daily) (4 × 6 week cycles). RESULTS: Median follow-up 67.7 months; OUTCOME: Overall survival, progression-free survival, and objective response were superior in the nivolumab plus ipilimumab group than in the sunitinib group, and nivolumab plus ipilimumab had a higher point estimate of 2-year conditional overall survival beyond 3 years compared with sunitinib. Conditional progression-free survival and point estimates of response were also higher for nivolumab plus ipilimumab than for more than 3 years [74,75]. The results suggest that a durable clinical benefit was observed at 5 years with nivolumab plus ipilimumab compared with sunitinib.

Thus, based on the relevant clinical trial data in recent years (Table 3), the current first-line treatment modalities for renal cell carcinoma, especially clear cell renal cell carcinoma, are mostly based on targeted immunotherapy or dual immunotherapy modalities. Although the mTOR and VEGFR pathways dominate in the management of renal cancer, the combination of VEGFR and mTOR inhibitors with ICI and geodetics improved the prognosis of patients with renal cell carcinoma.

## Finding New Treatment Options

The advent of ICIs has been a major breakthrough in oncology treatment. The approval of combination immunotherapy for advanced ccRCC has changed the standard of care for first-line treatment, and its efficacy in specific populations has been superior to previous agents (such as sunitinib) in all respects. However, many patients in the unscreened population do not benefit at all or benefit less, suggesting that there is still room for progress with ICIs. Three elements are required for the successful induction of an anti-tumor adaptive immune response: antigen, adjuvant, and a suitable immune microenvironment. The tumor microbial environment simultaneously influences all three elements, making it a promising combination for ICI treatment.

In recent years, as the molecular biology of renal cell carcinoma has been studied in depth, the mechanisms of immune escape in renal cell carcinoma, including MHC, immunosuppressive cells and their secreted immunosuppressive cytokines, and signal transduction of apoptotic molecules, have been investigated in depth. There is no doubt that the tumor microenvironment has a significant impact on immune escape; and whether the tumor environment can be altered so that cold tumors that do not respond to immunotherapy can be transformed into hot tumors that do respond to immunotherapy will be one of the next therapeutic directions.

As a special form of cancer cell death, ICD (immunogenic cell death) has the ability to alter the tumor microenvironment to a certain extent. When ICD occurs, certain molecules with immune effects are produced, which act in the tumor microenvironment and enhance the immunogenicity of the tumor.

## Immunogenic Cell Death

### Characteristics

ICD is a form of cell death that elicits an adaptive immune response in an immunocompetent environment [85], can induce adaptive immunity against dead cell antigens [86], and exposes, through the release of tumor-associated antigen (TAA) and tumor-specific antigen (TSA) “danger signals” to stimulate the body’s immune system to generate an immune response [87,88]. Certain stressors can trigger ICD, including endoplasmic reticulum stress, endogenous signals such as apoptosis, chemotherapeutic agents, chemotherapies such as lytic viruses, and physical therapies such as photodynamic and radiation therapy [85,89]. Indeed, there are established cancer treatments, including radiotherapy, specific chemotherapies, and some targeted therapeutic agents, which are immune responses that are involved in targeting cancer cells by triggering ICD and thus treatment [90,91].

ICD is characterized by the release and/or increased expression of danger-associated molecular patterns (DAMPs), precursor antigenic inflammatory cytokines, and inflammatory mediators [89]. Of these, DAMPs are key elements of ICD and are usually isolated within living cells, normally dormant, and when activated, they are translocated to the cell surface or secreted extracellularly, thus participating in the adaptive immune response of the tumor [92].

### Classification of DAMPs

DAMP can be divided into three main subgroups based on the phase and site of their localization/release.

### DAMP exposed to the plasma membrane, e.g., Calreticulin (CRT) and heat shock proteins (HSP70, HSP90)

**Figure 2:**
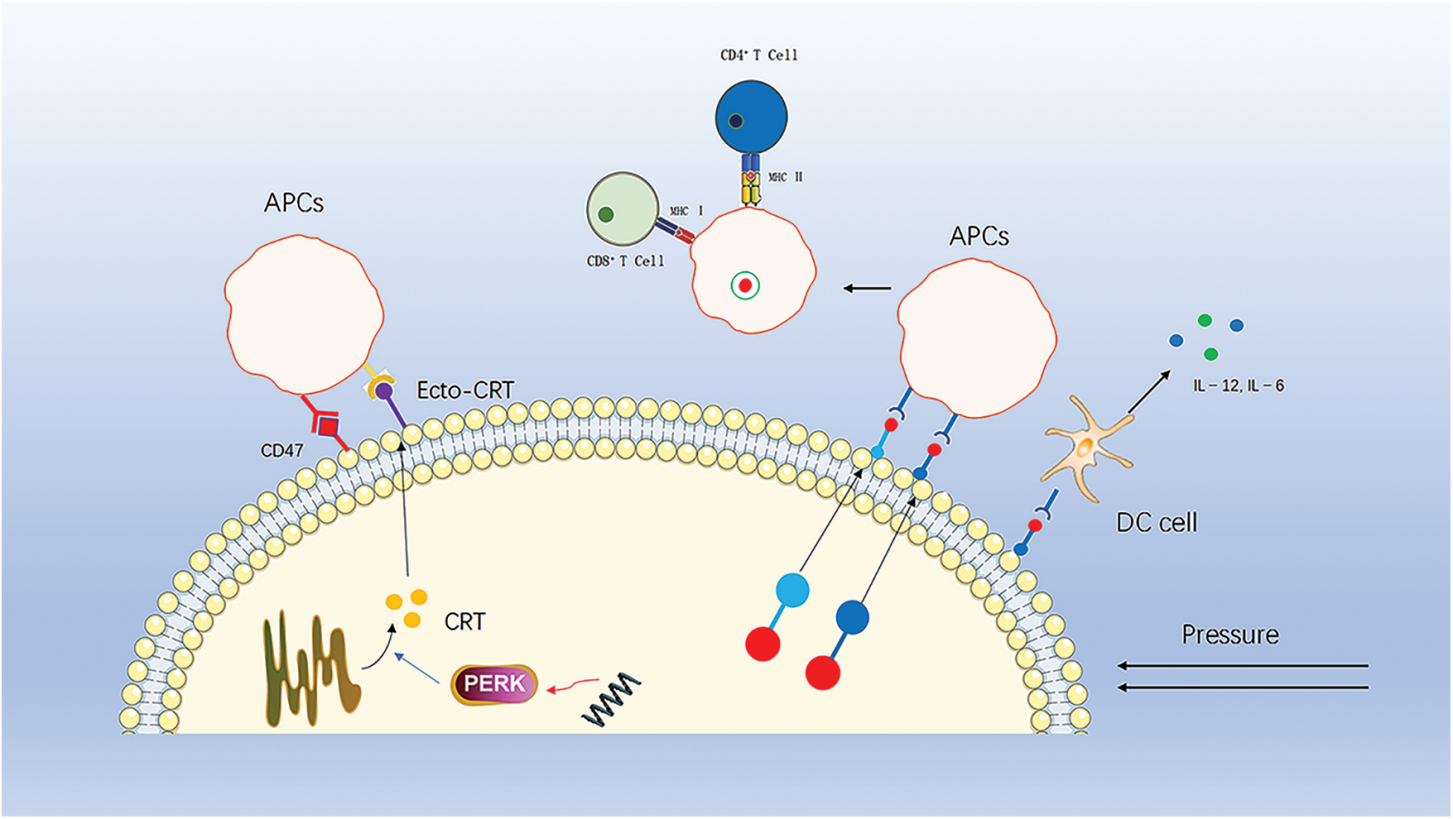
CRT translocates from the endoplasmic reticulum to the cell membrane to recruit APCs, a process that is inhibited by downregulation of perk and by CD47 on the cell surface. HSP70 and HSP90 carry tumor antigenic peptides that translocate to the cell membrane, recruit APCs and stimulate DC cells to secrete pro-inflammatory factors.

CRT is normally found in the endoplasmic reticulum (ER). Prior to ICD induction and cell membrane disruption, through endoplasmic reticulum stress, CRT translocates and is exposed to the cell membrane where it acts as a “eat me” signal for phagocytosis [93], and CRT on the cell membrane surface (ecto-CRT) enhances the immunogenic recognition and phagocytosis of dead cancer cells by APCs (Fig. 2).

This process is one of the key elements of ICD-driven anticancer immunity [86,94]. The immune effects of ecto-CRT can be antagonized or inhibited by several molecules. For example, small interference RNA (siRNA)-mediated downregulation of PERK inhibits eIF2a phosphorylation and ER stress, thereby suppressing CRT exposure. In addition, the anti-phagocytic CD47, a “don’t eat me” signal, also antagonizes the action of ecto-CRT [95]. When high levels of CRT are present on the surface of tumor cells, the expression of CD47 increases accordingly. Thus, increasing plasma membrane exposure to CRT while blocking or antagonizing CD47 is a strategy to induce ICD and enhance antitumor therapy.

HSP is a family of highly conserved molecular chaperone proteins with multiple chaperone functions [96]. Under stressful pressure, HSP are highly expressed and are another important signaling molecule mediating the immunogenic death of tumor cells. HSP70 and HSP90 carry tumor antigenic peptides that are transferred to the cell membrane and released into the tumor microenvironment, ultimately activating CTL cells, Th cells and DC cells [97–100]. Meanwhile, HSP70 also promotes DC cells to secrete pro-inflammatory factors, further enhancing the immune response [101].

### Extracellularly secreted DAM, e.g., High Mobility Group Box 1 (HMGB1)

**Figure 3:**
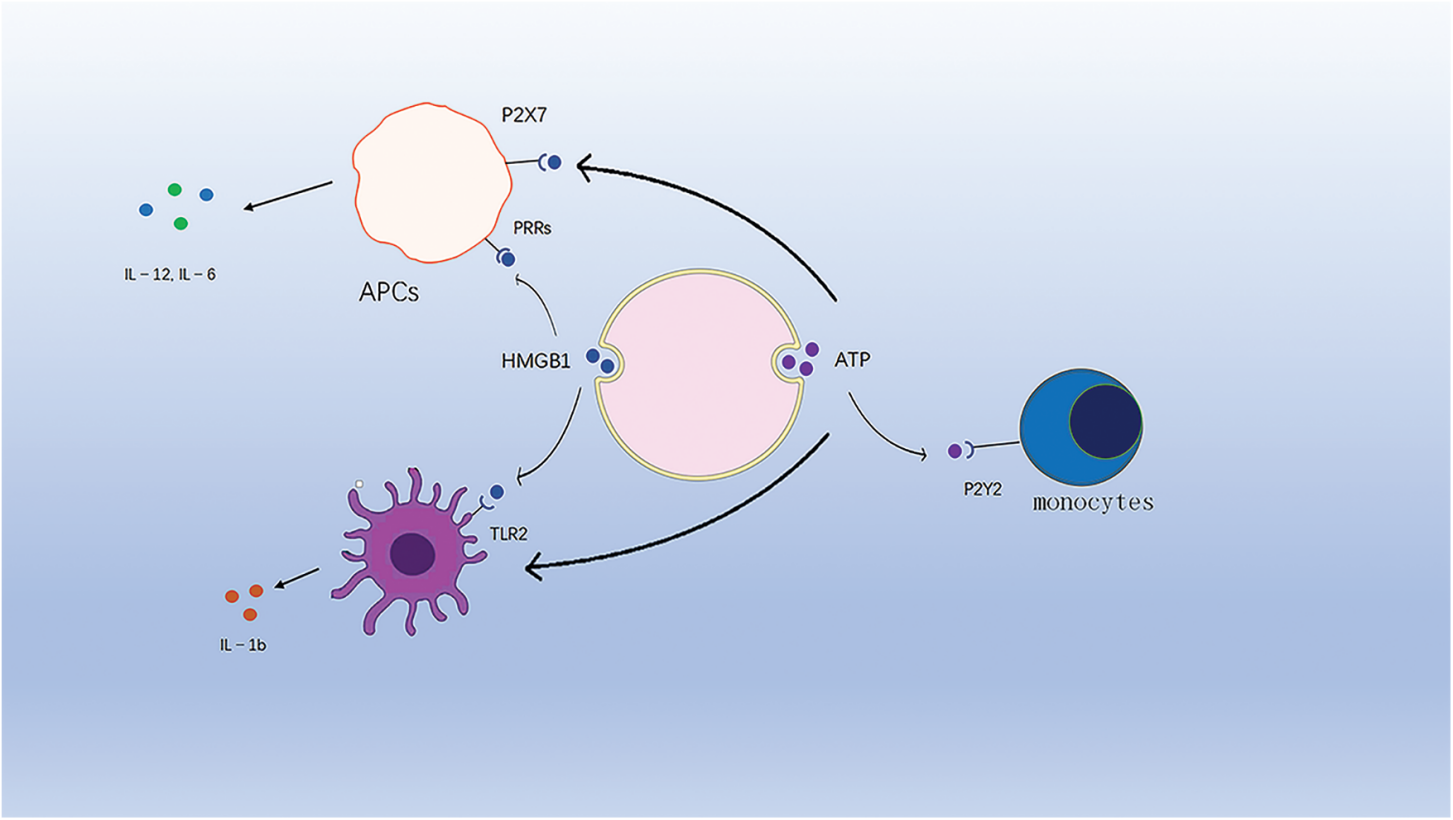
ATP binds to purinergic receptors on monocytes and stimulates their phenotypic maturation. HMGB1 binds to TLR4 to activate the corresponding signaling pathway. HMGB1 and ATP synergistically induce IL-1b release from DC cells.

HMGB1, proven to be a tumor suppressor, is normally responsible for DNA organization and transcriptional regulation. In the occurrence of ICD, HMGB1 is released from the cells to the extracellular compartment. Binding to PRRs, advanced glycosylation end-product-specific receptors (AGER or RAGE) and toll-like receptor 4 (TLR4) expressed on the surface of cells of myeloid origin activates the corresponding signaling pathways and promotes the immune response [102,103] (Fig. 3).

In addition, another study showed that HMGB1 synergistically induced IL-1b release from dc with ATP and that HMGB1-specific antibodies eliminated the ability of dc to contact dying tumor cells to produce IL-1b [104].

### DAMP is released as a final degradation product, e.g., ATP

In ICD, ATP is released extracellularly by dying tumor cells and acts as an endogenous danger signal. The release of ATP involves a series of complex processes, such as the activation of autophagy, apoptosis, and lysosomal cytosolic action, prior to cell death [105]. Extracellular ATP released from tumor cells is involved in the recruitment and activation of APCs. ATP released extracellularly binds to purinoceptors on APCs, stimulating their phenotypic maturation and mediating strong chemotaxis [106], and is also involved in mediating the formation of pro-inflammatory cytokines [107].

### Platinum-based immunogenic agents

Previous studies have shown that certain chemotherapeutic agents can trigger ICDs, including adriamycin and anthracyclines, epirubicin, and idarubicin [108], which are used to treat leukemia, breast cancer, gastric cancer, and lymphoma. In recent years, platinum-based drugs have become among the most widely used chemotherapeutic agents in clinical practice. Platinum-based drugs that are commonly used today include cisplatin, carboplatin, and oxaliplatin.

As the first platinum-based chemotherapeutic agent approved for use, the first generation platinum-based drug cisplatin was approved for the treatment of testicular and ovarian cancer in 1979 and has since been widely used in a variety of cancers [109,110]. Cisplatin exerts cytotoxic effects on DNA by binding to DNA as a result of the product of its conjugation with water. The second and third generation platinum drugs such as carboplatin, nedaplatin, and oxaliplatin have made improvements for the toxic side effects and drug resistance of cisplatin, which also allow them to be widely used in the treatment of various cancers [111–114].

Platinum-based chemotherapeutic agents, such as oxaliplatin, have contributed to the treatment of a variety of advanced tumors. In the treatment of advanced colorectal cancer, several clinical trials have shown that oxaliplatin combined with raltitrexed has an effective rate of 16% to 54%, a median PFS of 4 to 10.3 months and a median overall survival time (OS) of 9 to 14.8 months in the treatment of advanced colorectal cancer [115].

Despite the satisfactory success of platinum-based drugs and the development of various new small-molecule platinum-based drugs, the performance of platinum-based drugs has been disappointing for the treatment of kidney cancer, with clinical trials of oxaliplatin in patients with RCC showing little efficacy of platinum-based chemotherapy drugs. This is because oxaliplatin activity depends on the cellular uptake of the drug, which is mediated by organic cation transporter protein 2 (OCT2). However, in contrast to normal renal tissue, OCT2 expression appears to be downregulated in RCC samples [116]. This makes it necessary to find new ways to improve the efficacy of platinum-based drugs.

### Combination of platinum drugs with PD-1/PD-L1 inhibitor drugs

Clinical trials of platinum-based drugs in combination with PD-1/PD-L1 drugs are ongoing for the treatment of certain tumors, particularly non-small-cell lung cancer (NSCLC), a devastating disease with a 5-year survival rate of less than 2% 1 year after diagnosis [117]. Since the 1980s, platinum-based chemotherapy has been the treatment of choice for NSCLC, and when ICI was introduced, researchers tried to use it alone in the treatment of NSCLC without satisfactory results [118]. However, several recent trials have established the role of PD-1/PD-L1 inhibitors as first-line therapy in combination with platinum-based chemotherapy (Table 4).

**Table 4:** Randomized controlled trial of platinum-based combination of anti-PD-1/PD-L1 and chemotherapy for ES-SCLC

Susbstances	n	Patients	Results			Adverse events	References
IMpower133 phase = 3 Atezolizumab + carboplatin/ etoposide with atezolizumab maintenance *vs*. 0Carboplatin/etoposide	403	Extensive-stage SCLC and no prior systemic therapy	PFS(median) 5.2 *vs*. 4.3	OS (median)12.3 *vs*. 10.3	ORR(%) 60 *vs*. 64	Grade 3 or 4 immune-related adverse events occurred in 8.1% of patients in atezolizumab and 2.6% of patients in placebo.	[119]
CASPIAN phase = 3 Durvalumab + platinum/etoposide with durvalumab maintenance *vs*. platinum/etoposide	537	Eligible patients were aged 18 years or older (20 years in Japan) and had treatment-naive, histologically or cytologically documented ES-SCLC, with a WHO performance status of 0 or 1	PFS(median) 5.1 *vs*. 5.4	OS (median) 12.9 *vs*. 10.5	ORR(%) 68 *vs*. 58	Grade 3 or 4 immune-mediated AEs were reported in 5% of patients who received durvalumab.	[120]
KEYNOTE604 phase = 3 Pembrolizumab + platinum/etoposide with pembrolizumab maintenance *vs*. platinum/etoposide	453	Key eligibility criteria were age ≥18 years; histologically or cytologically confirmed SCLC not previously treated with systemic therapy	PFS(median) 4.5 *vs*. 4.3	OS (median) 10.8 *vs*. 9.7	ORR(%) 71 *vs*. 62	Grade 3 or 4 immune-related adverse events occurred in 76.7% of patients in the Paborizumab group and 74.9% of patients in the placebo group.	[121]

In the Impower 133 trial, for example, the investigators randomly assigned 403 patients with extensive-stage SCLC who had not previously received systemic therapy with the anti-PD-L1 monoclonal antibody atezolizumab or placebo arm and received concurrent carboplatin and etoposide chemotherapy for four cycles. Atezolizumab or placebo was continued as maintenance therapy until disease progression or adverse toxicity. The primary endpoint of median OS was 12.3 months (95% CI, 10.8–15.8) in the atezolizumab group and 10.3 months (95% CI, 9.3–11.3) in the placebo group, with a 24% reduction in HR for death (HR, 0.76; 95% confidence interval, 0.60–0.95) [119]. The other two trials were similar to this trial, and it can be seen that the combination of platinum with PD-1/PD-L1 inhibitors was successful in the treatment of NSCLC. This provides ideas for the treatment of other tumors.

Following the success of trials with a combination of drugs, researchers have further explored the mechanism of the combination. Platinum-based drugs have both positive and negative modulatory effects on the immune system. Platinum-based chemotherapy enhances the immune response through ICD induction, enhanced T-cell activation, upregulation of tumor-killing immune cell activity, downregulation of the immunosuppressive microenvironment and enhanced killing by CTLs [122,123]. Positive immunomodulation increases the sensitivity of tumor cells to PD-1/PD-L1 inhibitors. In contrast, platinum-based chemotherapies upregulate PD-L1 expression in certain preclinical tumor models, which can be blocked by PD-1/PD-L1 inhibitors [124]. Furthermore, different types of platinum-based chemotherapy have different immunomodulatory properties. Different doses of platinum-based drugs may exert different mechanisms of action when combined with PD-1/PD-L1 inhibitors.

## Perspectives on the Treatment of Kidney Cancer

The success of combination drug therapy for NSCLC has opened new avenues for oncology treatment. Although no studies have yet been published on the treatment of kidney cancer, it is foreseeable that new combination drug strategies could be available in the future as they are being studied more intensively as one of the beneficiaries of PD-1/PD-L1 inhibitors. Platinum drugs are also being studied; unlike traditional platinum drugs, these nanoscale particles can reach the respective sites more efficiently with the help of a carrier, possessing higher efficacy and lower side effects. These novel nanoparticles exhibit significant aggregation at the tumor site, suggesting that they can be used in a broad dose when combined with PD-1/PD-L1 inhibitors [125]. Although these mechanisms indicate new therapeutic directions, new combination therapies need to be explored.

## Conclusion

The incidence and mortality of kidney cancer, a common tumor of the urinary system, are increasing annually. In this article, we summarize the development of therapeutic tools for clear cell carcinoma of the kidneys. As researchers study cellular pathways and deepen their understanding of tumors, from macroscopic to microscopic, the treatment of kidney cancer is involving intracellular molecules. In the past decade, the emergence of ICIs marked the formal entry of tumor therapy into the immunotherapy phase, and anti-PD-1/PD-L1 antibodies have become one of the most widely used anti-cancer therapies, which are used in the first-line treatment of kidney cancer. A large number of clinical trials have shown that ICIs are superior to previously used drugs in terms of PFS, OS, OR, and many other aspects. However, the ICIs are not as effective in unscreened populations. Therefore, to maximize the potential of ICIs to bring benefits to more patients, we considered whether there are other mechanisms of cell death that could be involved in the combination of drugs to compensate for the disadvantages of ICIs. ICD, as a cell death that induces an immune response, is expected to break the immunosuppressive tumor microenvironment, initiate a T cell-mediated adaptive immune response, and block the immune escape mechanism of the tumor, thus promising a long-term tumor-suppressive effect. The ICD has made some achievements in the treatment of other cancers, while expanding the horizon for the treatment of kidney cancer. Platinum-based drugs, which are one of the most widely used chemotherapeutic agents, have been shown to trigger ICDs. ICIs have been used in combination with platinum-based chemotherapeutic agents for the treatment of NSCLC, with satisfactory results. Further studies have found that PD-1/PD-L1 inhibitors complement platinum-based drugs, providing a 1+1 effect that is greater than 2. As research has progressed, breakthroughs have been made with platinum drugs, and newly developed nanoscale platinum drugs can synergize with PD-1/PD-L1 inhibitors. However, there are some other problems with the application of ICD, for example, there seems to be more DAMPs, and when cancer cells die they release various metabolites including ATP, spermidine, one of them, can mediate local anti-inflammatory effects and also stimulate immune response, suggesting that it may also belong to DAMPs [126]. In addition to ICD, various newly discovered mechanisms of cell death such as ferroptisis, pyroptosis, and cuproptosis also possess great potential, and after the mechanisms are further elucidated and relevant markers are discovered, breakthroughs in the treatment of kidney cancer may also be made again. It is undeniable that the treatment of kidney cancer is still a difficult problem. Combination therapy is still on the way to be explored, and more potential mechanisms are waiting to be explored to develop better combination drug strategies.

## Abbreviations

RCC: Kidney cancer
ccRcc: Renal clear cell carcinoma
DC: Dendritic cell
MAGE: Melanoma associated antigen
APC: Antigen presenting cell
CTL: Cytotoxicity T lymphocyte
HLA: Human leukocyte antigen
ICI: Immune checkpoint inhibitors
IL: Interleukin
IFN: Interferon
TNF: Tumor necrosis factor
VEGF: Vascular endothelial growth factor
TKI: Tyrosine kinase inhibitor
MTOR: Mammalian target of rapamycin
ACD: Accidental cell death
RCD: Regulation of cell death
ICD: Immunogenic cell death
DAMPs: Danger associated molecular patterns
CRT: Calprotectin
ER: Endoplasmic reticulum
HSP: Heat shock protein
PERK: Protein kinase-like endoplasmic reticulum kinases
HMGB1: High Mobility Group Box 1
PRRs: Pattern recognition receptor molecules
GSDMB/D/E: Gasdermin B/D/E
GSH: Glutathione
